# Myeloid Derived Suppressor Cells (MDSCs) Are Increased and Exert Immunosuppressive Activity Together with Polymorphonuclear Leukocytes (PMNs) in Chronic Myeloid Leukemia Patients

**DOI:** 10.1371/journal.pone.0101848

**Published:** 2014-07-11

**Authors:** Cesarina Giallongo, Nunziatina Parrinello, Daniele Tibullo, Piera La Cava, Alessandra Romano, Annalisa Chiarenza, Ignazio Barbagallo, Giuseppe A. Palumbo, Fabio Stagno, Paolo Vigneri, Francesco Di Raimondo

**Affiliations:** 1 Department of Clinical and Molecular Biomedicine, section of Hematology, Ferrarotto Hospital, University of Catania, Catania, Italy; 2 Department of Drug Sciences, section of Biochemistry, University of Catania, Catania, Italy; 3 Department of Pediatric and Medical Sciences, University of Catania, Catania, Italy; University of Catania, Italy

## Abstract

Tumor immune tolerance can derive from the recruitment of suppressor cell population, including myeloid derived suppressor cells (MDSCs), able to inhibit T cells activity. We identified a significantly expanded MDSCs population in chronic myeloid leukemia (CML) patients at diagnosis that decreased to normal levels after imatinib therapy. In addition, expression of arginase 1 (Arg1) that depletes microenvironment of arginine, an essential aminoacid for T cell function, resulted in an increase in patients at diagnosis. Purified CML CD11b+CD33+CD14-HLADR- cells markedly suppressed normal donor T cell proliferation in vitro. Comparing CML Gr-MDSCs to autologous polymorphonuclear leukocytes (PMNs) we observed a higher Arg1 expression and activity in PMNs, together with an inhibitory effect on T cells in vitro. Our data indicate that CML cells create an immuno-tolerant environment associated to MDSCs expansion with immunosuppressive capacity mediated by Arg1. In addition, we demonstrated for the first time also an immunosuppressive activity of CML PMNs, suggesting a strong potential immune escape mechanism created by CML cells, which control the anti-tumor reactive T cells. MDSCs should be monitored in imatinib discontinuation trials to understand their importance in relapsing patients.

## Introduction

The tyrosine kinase inhibitors (TKIs), imatinib (IM), nilotinib and dasatinib have changed the course of the chronic myeloid leukemia (CML) [1] but these drugs are not able to eradicate CML clone [2]. However, a study with patients discontinuing IM has shown that 41 percent of the patients stopping treatment while in complete molecular response (CMR), remained in CMR at 12 months of follow-up [3]. In these patients, it is possible that the immune system plays a role in maintaining complete remission. This observation, together with the finding of BCR/ABL transcripts in some healthy individuals [4], could support the hypothesis that in some patients the immune system may exert an immune surveillance against leukemic cells, while a suppression of this system may create a permissive environment for development and progression of disease. Indeed, immunotherapy in combination with TKIs is currently tested in clinical trials for treatment of CML [5], [6].

CD4+ T cells are central components of effective immune response against cancer cells. This T cells subset appear to be anergic to the leukemia cells in CML patients [7], [8] and show decreased expression of the TCR-ζ chain compared with T cells of healthy individuals [9]. In addition, CML cells produce large amounts of IL-10 [7], [10], a cytokine which is known to suppress cytokine expression by T cells [11]. Consequently, it is important the identification of cell types and mechanisms responsible for the induction and/or maintenance of T cell anergy in CML patients.

In many solid tumors, a subpopulation of myeloid cells defined as Myeloid Derived Suppressor Cells (MDSCs) is supposed to play a central role providing a favorable microenvironment in which transformed cells can proliferate, acquire new mutations, expand and evade host immunosurveillance [12], [13]. MDSCs represent a phenotypically heterogeneous population of myeloid cells at different stages of maturation [12], that are able to suppress tumor-specific T cell response through the induction of T cell anergy and the development of T-regulatory cells (T-reg) [14]. Two important subsets of human MDSCs have been reported: granulocytic MDSCs (Gr-MDSCs) with a CD11b+CD33+CD14-HLADR- phenotype and monocytic MDSCs (Mo-MDSCs) that are CD14+HLADR- [15]–[19].

MDSCs inhibit T cell activation and function by producing ROS and nitrogen species which down regulate or dissociate the CD3-associated ζ chain from the T cell receptor (TCR) thus inhibiting T cells function. In addition, MDSCs and in particular Gr-MDSCs produce arginase I (Arg1) and deplete their environment of L-arginine and L-cysteine, amino acids required for T cell activation and proliferation [20]. Finally, MDSCs promote expansion of T-reg cells through production of IL-10, TGF-β and INFγ [21], [22].

Since tumor cells in CML are immature and of myeloid origin their role as potential MDSCs is of interest to investigate.

## Materials and Methods

### Patients and sample collection

To participate in this study, all participants signed a written informed consent approved with the study (34/2013/VE) by the local ethical committee (Azienda ospedaliero Universitaria Policlinico-Vittorio Emanuele). After written informed consent, samples were collected from CML patients and age-matched healthy donors (HD) at Division of Hematology, AOU Policlinico – OVE, University of Catania. CML patients at diagnosis and during TKIs treatment were enrolled in this study. During treatment, all patients were followed with a monthly CBC count, molecular evaluation of the BCR/ABL transcript every 3 months and cytogenetic evaluation every 6 months, according to ELN guidelines.

### Flow cytometry analysis of MDSCs phenotype

The amount of MDSCs was evaluated in peripheral blood (PB) of 20 HD, 19 CML patients at diagnosis and 18 during IM therapy (13 of whom collected at diagnosis too). Analysis of MDSCs was performed by multicolor FACS analysis using the following antibody (Beckman Coulter): CD14 PC5 (clone RMO52), HLADR ECD (clone IMMU-357), CD11b FITC (clone bear-1), CD33 PE (clone D3HL60, 251) and their respective isotype controls. Briefly, 1×10^6^ cells were stained with 10 µl of each of the above listed Abs and incubated for 20 minutes in the dark at room temperature. After lysing red cells with ammonium chloride, cells were analyzed by flow cytometer (Cytomics FC 500, Beckman Coulter) and analysis was performed using CXP Analysis software. Using sequential gating strategy, Gr-MDSCs cells were identified as cells CD11b+CD33+CD14-HLADR-, while the Mo-MDSCs as CD14+HLADR-. The results were expressed both as percentage and absolute number. The amount of MDSCs was also evaluated in bone marrow (BM) samples of five CML patents at diagnosis and compared to PB from the same patients.

In 12 CML patients we also evaluated the frequency and phenotype of T-reg (CD4+CD25^high^, Foxp3+) at diagnosis and during therapy with IM. For the evaluation of T-reg, 1×10^6^ cells were incubated with 10 µl of following mAbs (Beckman Coulter): CD4 Pc5 (clone 13b8.2) and CD25 PE (clone B149.9) for 20 minutes in the dark at room temperature. Subsequently, intracellular Foxp3 FITC (clone PCH101 eBioscence) and relevant isotype control staining was performed according to the manufacturer's instructions. T-reg was enumerated both as percentage and absolute number.

### Investigation of BCR/ABL transcripts in granulocytic and monocytic MDSCs subsets

Gr-MDSCs and Mo-MDSCs were isolated from peripheral blood mononuclear cells (PBMNCs) by the magnetic separation (CD14-negative and CD66b-positive selection, *StemCell Thechnologies* and Anti-HLA-DR MicroBeads and CD14 MicroBeads, *Miltenyibiotec*). The purity and viability was more than 90% as tested by flow cytometry. After RNA extraction and reverse transcription, we performed quantitative real-time PCR using a 7900HT Fast Real-Time PCR System (Applied Biosystems) with the BioQuant p210 BCR-ABL kit (Biodiversity). For each sample the fusion gene, either b2a2 or b3a2, and the housekeeping gene ABL transcripts were detected. The cycle threshold values for BCR-ABL and the control gene were determined in duplicate for each sample.

### RNA extraction and qRT-PCR

Most samples from patients at diagnosis were collected before any treatment but 9 had been treated with hydroxyurea (HU) for 2–6 days prior to sample collection. Cells from PB of CML patients and HD were obtained collecting the buffy coat layer of cells from the whole blood and lysing the residual red cells. Gr-MDSCs were obtained from freshly isolated PBMNCs as described above. Cell purity was determined by flow cytometry and was >90%.

After RNA extraction and reverse transcription, Arg1 mRNA expression was assessed by TaqMan Gene Expression, Applied Biosystem and quantified using a fluorescence-based real-time detection method by 7900*HT* Fast Start (Applied Biosystem). For each patient, the relative expression level of Arg1 mRNA was normalized using ABL and GAPDH as invariant controls.

### ELISA for Arg1

By using a specific ELISA test, according to the manufacturer's recommendations, we measured Arg1 protein in HD (n = 10) and CML sera at diagnosis (n = 15) and followed during IM therapy (n = 10) (BioVendor Laboratory Medicine, Candler, NC, USA). Absorbance at 450 nm was evaluated by a spectophotometer and Arg1 concentration was calculated on the basis of standard curve.

### Isolation of polymorphonuclear leukocytes (PMNs)

To isolate HD and CML PMNs, the pellet obtained after centrifugation of PB on Ficoll, containing erythrocytes and polymorphonuclear leukocytes (PMNs), was subjected to hypotonic lysis (155 mM NH_4_ Cl, 10 mM KHCO_3_, 0.1 mM EDTA, pH 7.4) for 15 minutes on ice [23]. After washing, cell purity and viability were checked by flow cytometry and microscopy. PMNs showed a purity and viability of more than 90%.

### Western blot analysis

Western Blot Analysis was performed according to the manufacturer's recommendations [24]. The antibody directed against the human Arg1 was obtained from Santa Cruz Biothecnology (CA, USA). An anti-mouse antibody against actin (Sigma, St. Louis, MO, USA) was used to assess equal loading. The blots were scanned, and the optical density of the bands was measured using Scion Image software (New York, NY).

### Arginase enzymatic assay

Arginase activity was measured in cell lysates as follows. Briefly, Gr-MDSCs and PMNs from fresh blood were lysed with 0.5% Triton X-100, 25 mM Tris (tris(hydroxymethyl)aminomethane)–HCl, pH 7.5; 10 mM MnCl_2_ was added to this lysate, and the enzyme was activated by heating for 10 minutes at 56°C. Arginine hydrolysis was conducted by incubating the lysate with 0.5 M L -arginine (pH 9.7) at 37°C for 1 h. The reaction was stopped with 900 ml of H_2_SO_4_ (96%)/H_3_PO_4_ (85%)/H_2_O (1/3/7, v/v/v). The urea concentration was measured at 540 nm after the addition of α-isonitrosopropiophenone followed by heating at 95°C for 30 min. One unit of enzyme activity is defined as the amount of enzyme that catalyzes the formation of 1 µMol of urea in 1 h at 37°C.

### T cell functional assays

T cells were isolated by Ficoll-Hypaque density-gradient centrifugation and purified using T-cells enrichment columns (R&D Systems) with a purity and viability of more than 90% as tested by flow cytometry. Cells were stimulated with 5 mg/mL phytohemagglutinin (PHA; Sigma) per well and incubated for 72 hours post-stimulation. Cells were cultured in RPMI-1640 medium with 10% FBS and 1% penicillin-streptomycin. Sera from CML patients at diagnosis and HD at different concentration (10%, 20%) [25] with or without 10 µM Nor.NOHA (Sigma Aldrich) were added at the time of plating and PHA addition.

In functional assays, T cell proliferation was measured by carboxyfluorescein succinimidyl ester (CFSE) staining. Proliferation rate of lymphocytes was determined by assessing the reduction of the intensity of the fluorescent cell permeable dye CFSE, which is retained in the cytosol through cellular esterases and equally distributed into both daughter cells during mitosis. For cell labeling, 5×10^5^ lymphocytes were incubated at 37°C for 20 min in 1 ml PBS containing 1 µM CFSE (BD Pharmingen). Cells were then stimulated with 5 mg/mL PHA and incubated for 72 hours prior to flow cytometry. T cell proliferation was measured by CFSE dilution.

The suppressive function of CML Gr-MDSCs and PMNs was measured by their ability to inhibit the proliferation of HD T cells in the following Suppression Assay: CFSE-labeled T cells were seeded in 96-well plates with CML Gr-MDScs or PMNs at 4∶1 ratio [26]. T cell proliferation was induced by PHA stimulation [27]. Controls included a positive T cell proliferation control (T cells plus PHA) and a negative one (T cells only). Every condition was analyzed by flow cytometry for T cell proliferation after three days.

Separation of Gr-MDSCs from frozen samples causes a significant decrease in MDSCs viability and function [28]; therefore, all above described functional assays were performed using fresh blood.

### Statistical analysis

Statistical analyses were made with Prism Software (Graphpad Software Inc., La Jolla, CA, USA). The data is expressed as mean ± SEM. Statistical analysis was carried out by paired Student's t-test or ANOVA test. A p value <0.05 was considered to indicate a statistically significant difference between experimental and control groups.

## Results

### Gr- and Mo-MDSCs are increased in CML patients at diagnosis

**Figure 1:**
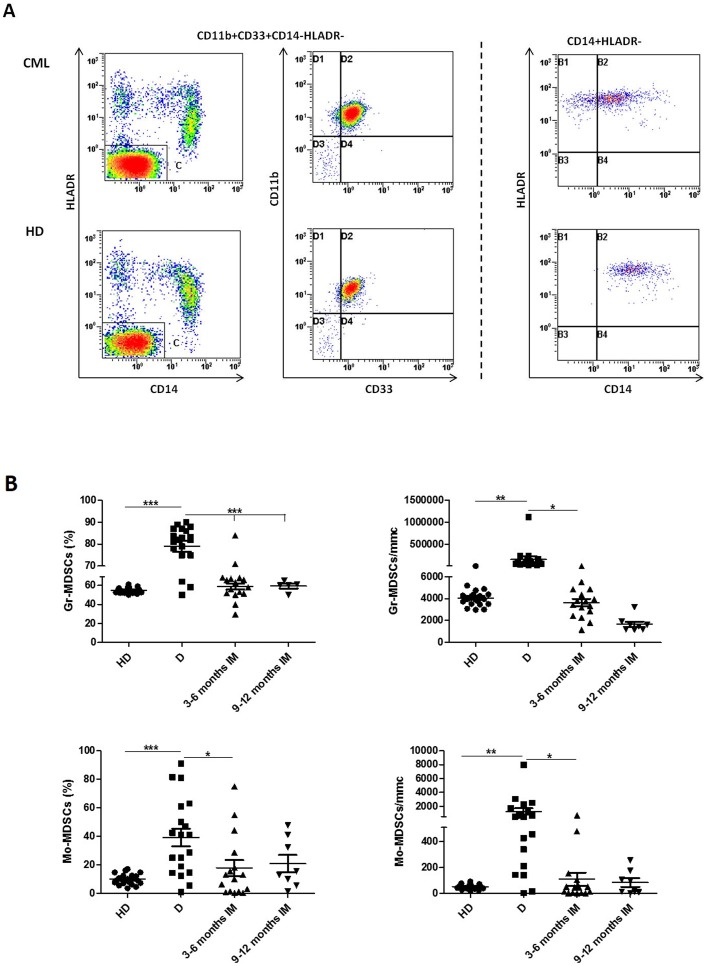
Flow cytometry analysis of circulating Gr-MDSCs and Mo-MDSCs cells in PB from HD and CML patients at diagnosis (D) and during IM therapy. A: Representative data from one HD and one CML at diagnosis. Flow cytometry analysis was performed with gates set on either CD11b+CD33+CD14-HLADR- or CD14+HLADR-cells populations. B: Figures show the quantitative data for Gr-MDSCs and Mo-MDSCs. Results are expressed either as percentage or absolute number. Statistically significant differences between groups are indicated by P-value in the figures.

The proportions of CD11b+CD33+CD14-HLADR- (Gr-MDSCs) and CD14+HLADR- (Mo-MDSCs) cells in the PB of CML patients at diagnosis and during IM therapy were evaluated using flow cytometry. Representative flow cytometry results for one patient and one HD are shown in fig.1A. The percentage of Gr-MDSCs and Mo-MDSCs cells was significantly elevated in patients at diagnosis compared to HD (79,1±10% vs 54,6±3% for Gr-MDSCs, p<0.0001 and 37,3±17,2% vs 9,8±3,8% for Mo-MDSCs, p<0.001) (fig.1B). The results expressed as absolute number showed also a significant difference between the two groups (74071±977 vs 4071±291 for Gr-MDSCs and 1280±872 vs 57±18 for Mo-MDSCs; p<0.001). Both Gr-MDSCs and Mo-MDSCs subpopulations, measured as percentage and as absolute number, returned to normal levels during treatment with IM (fig 1B). MDSCs count on BM samples did not show differences with the corresponding PB (data not shown). Clinical data of patients enrolled at diagnosis are reported in table 1. The percentage of MDSCs did not correlate neither with age, nor with leukocytosis or Sokal risk. In addition, no correlation was observed between MDSCs and the response to IM. Our set comprises only 3 patients who were considered resistant to IM and they showed very high levels of MDSCs, although not the highest observed.

**Table 1:** Clinical characteristics of CML patients recruited at diagnosis.

Patients	Gender	^1^Age	BCR/ABL transcript levels	WBC (10^3^/µL)	HGB (g/dL)	PLT (10^3^/µL)	LDH (mg/dL)	Liver (cm)^2^	Spleen (cm)^2^	Sokal risk group	Gr-MDSCs (%)	Mo-MDSC (%)
1	M	59	25,14	75,6	15,3	20	-	0	0	low	87	18,9
2	F	48	81,88	67,14	8,1	458	1043	2	14	high	86	41
3	F	71	48,16	70,5	9,2	338	2230	-	-	intermediate	89	46,8
4	M	70	349,51	71,2	13,5	370	715	0	0	intermediate	79	81,2
5	F	66	126,73	27,6	12,9	294	888	2	3	intermediate	82	12,4
6	M	38	65,61	28,8	13,6	232	-	0	0	low	83,4	28,5
7	M	21	126,51	144	14,2	107	1247	0	6	low	83,7	81,6
8	M	53	142,78	24,8	16,6	311	288	0	3	low	83	63
9	M	48	40,33	259,9	9,9	350	1074	7	14	intermediate	90	41
10	F	64	28,21	55	10,6	531	1147	0	2	intermediate	58,3	1
11	F	57	122,97	15,3	11,4	273	1124	2	1	low	88	61
12	M	36	56,24	136,6	10,8	208	1352	7	8	low	87	42,4
13	M	52	14,89	46	10,2	418	1820	0	2	low	78	25
14	F	58	150,04	122	10,7	361	1693	0	3	int	75	50
15	F	65	191,66	87	15,3	252	688	0	0	low	50	91,2
16	M	72	71,98	211,9	13	168	345	2	4	int	75	14,4
17	F	78	48,22	58,5	11,7	651	-	0	1	high	64	5,4
18	M	37	153,59	104,3	12,8	344	1635	0	0	low	82,5	2,8
19	M	69	62	85	12,2	320	260	0	0	low	83	25

Abbreviations: F, Female; M, male; WBC, white blood cells; HGB, hemoglobin; PLT, platelets; LDH, lactate dehydrogenase. PB: peripheral blood. Liver and spleen, cm below the costal margin. ^1^Age at diagnosis.

In order to evaluate whether MDSCs belong to the tumor clone or normal residual cells, in 3 patients we analyzed BCR/ABL expression in both CD11b+CD33+CD14-HLADR- and CD14+HLADR- subpopulation cells and all samples showed the oncoprotein expression in both the two subsets (data not shown).

**Figure 2:**
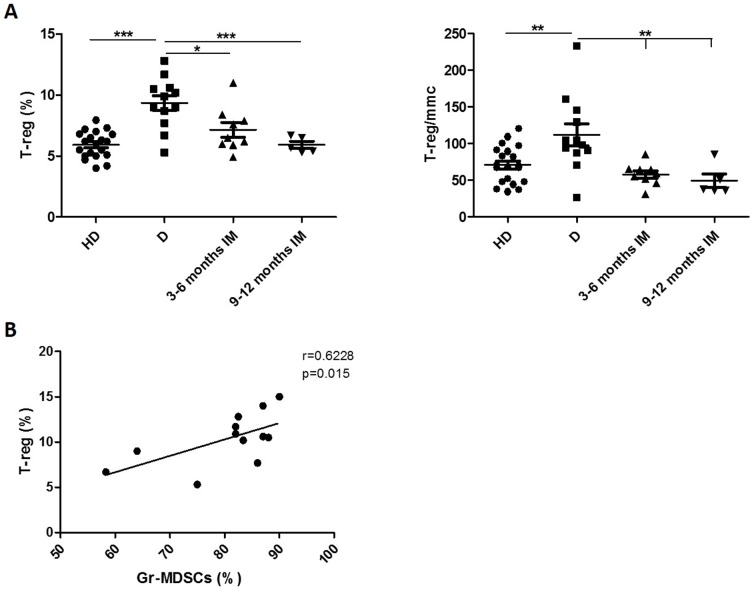
Frequency of circulating T-reg cells in PB from HD and CML patients at diagnosis and during IM therapy. **A**: Figures show the quantitative data expressed either as percentage or absolute number. Cytometric analysis was performed with gates set on CD4+ cells and the results presented as the percentage of CD25+Foxp3+cells in CD4+ cells. Statistically significant differences between groups are indicated by P-value in the figures. **B**: Correlation of the percentages of Gr-MDSCs cells and T-reg in PB from CML patients at diagnosis.

Since it has been reported that MDSCs may promote T-reg expansion, we also evaluate this lymphoid subpopulation and found that in these CML patients T-reg was significantly increased at diagnosis in respect to HD both as percentage (9,3±2% vs 5,9±0,8%; p<0.0001) and absolute number (111,7±51% vs 70,6±25,4%; p<0.001) and returned to normal levels during therapy with IM (fig.2A). In addition, at diagnosis, T-reg cells correlated with Gr-MDSCs (r = 0,6228; p = 0.01, fig.2B), but not with Mo-MDSCs (r = 0,3379; p = 0.14).

### CML patients have increased Arg1 expression and increased levels of circulating protein

**Figure 3:**
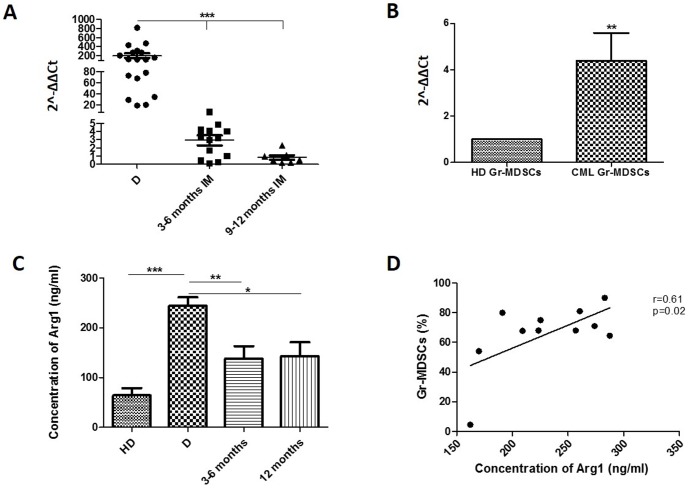
Increased Arg1 expression in CML patients at diagnosis. **A**: Arg1 mRNA expression in HD and CML patients at diagnosis (D) and during IM therapy assessed by real time PCR. Healthy donors (HD) vs D and D vs therapy: p<0.0001. **B**: Levels of Arg1 mRNA in Gr-MDSCs from HD and CML patients at diagnosis. The expression resulted higher in CML Gr-MDSCs in respect to HD (calculated value of 2∧-ΔΔCt in HD was 1; p<0.001). **C**: Arg1 concentration measured by ELISA. Circulating protein was increased in CML patients at diagnosis compared to HD (p<0.0001) and decreased during IM therapy (D vs 3–6 and 12 months: p<0.001 and p<0.05 respectively). **D**: Correlation of the percentage of Gr-MDSCs cells in PB and Arg1 levels in serum of CML patients at diagnosis.

The expression of the MDSCs associated molecule, Arg1, was assessed in leukocytes from HD and CML patients at diagnosis and during IM therapy. The relative expression of Arg1 in CML samples was 200 fold higher than HD (p<0.0001) (fig.3A). IM therapy reduced Arg1 expression to normal levels (diagnosis vs therapy: p<0.0001).

Since high Arg1 expression is characteristic for Gr-MDSCs, we investigated the mRNA levels in Gr-MDSCs obtained from freshly isolated PBMNCs by magnetic separation. Analyzing this MDSCs subpopulation in CML patients at diagnosis (n = 8) and HD (n = 4), we observed an increase of expression of about 4,3 fold (p<0.001) in CML Gr-MDSCs compared to HD (fig.3B).

Using an ELISA test, we measured the enzyme concentration in sera from HD and CML patients. The differences in Arg1 concentration between HD and CML patients at diagnosis was statistically significant (64±37,5 vs 243,9±64,3 ng/ml; p<0.0001) (fig.3C). After treatment, Arg1 level decreased at 3 and 12 months of IM therapy but it remained higher than HD.

We also found a significant correlation (r = 0,61; p = 0.02) between the percentage of Gr-MDSCs and Arg1 protein levels in the serum of patients at diagnosis (n = 11 patients) (fig.3D).

### Purified CML Gr-MDSCs and PMNs are immunosuppressive cells

Arg1 appears to be a major mediator of T cell suppression in both Gr-MDSCs and PMNs in cancer patients [27], [29], [30]. Therefore, especially in CML, it is important to compare Gr-MDSCs with autologous PMNs.

**Figure 4:**
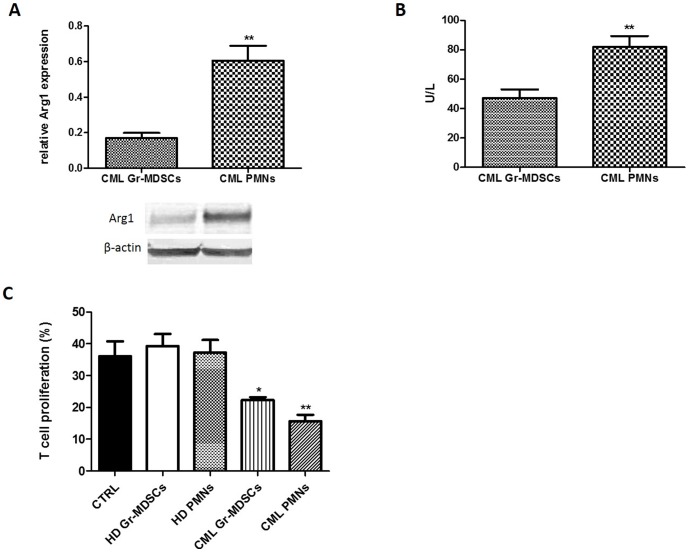
Gr-MDSCs and PMNs exert immunosuppressive activity in CML patients. Arg1 expression (**A**) and activity (**B**) were significantly increased in CML PMNs more than autologous Gr-MDSCs isolated from patients at diagnosis. For analysis of western blot the optical density of the bands was measured using Scion Image software. Results represent the means of three independent experiments; error bars denote SEM. (**C**) The percentage of T cell proliferation was significantly reduced when T cells were cultured with CML Gr-MDSCs (p<0.05) and CML PMNs (p<0.001), while Gr-MDSCs and PMNs from HD did not exert any suppressive activity. Mean frequency of CD3+ CFSE ^dim^ cells ± SEM from four independent experiments in duplicate is shown.

Evaluating Arg1 expression and activity in 8 patients at diagnosis, the protein levels resulted significantly higher in CML PMNs in respect to autologous Gr-MDSCs (p<0.001; fig.4A) so as enzymatic activity (47,2±15,3 U/L in CML Gr-MDSCs vs 81,8±19,9 U/L in CML PMNs; p<0.001) (fig.4B).

To verify whether both Gr-MDSCs and PMNs in CML patients could be immunosuppressive cells, we tested their ability to inhibit T cells proliferation. Cocultures were prepared using purified Gr-MDSCs and PMNs from HD and patients at diagnosis and CFSE-labeled T cells from HD. After 72 h from mitogen stimulation, the proliferation of T cells was inhibited of 13,8±1,5% and 20,5±3,5% respectively with CML Gr-MDSCs and CML PMNs compared with control (HD T cells plus PHA) (p<0.05 and p<0.001) while no suppressive effect on T cells was observed after incubation with HD Gr-MDSCs or PMNs (fig.4C).

### CML serum has immunosuppressive activity

**Figure 5:**
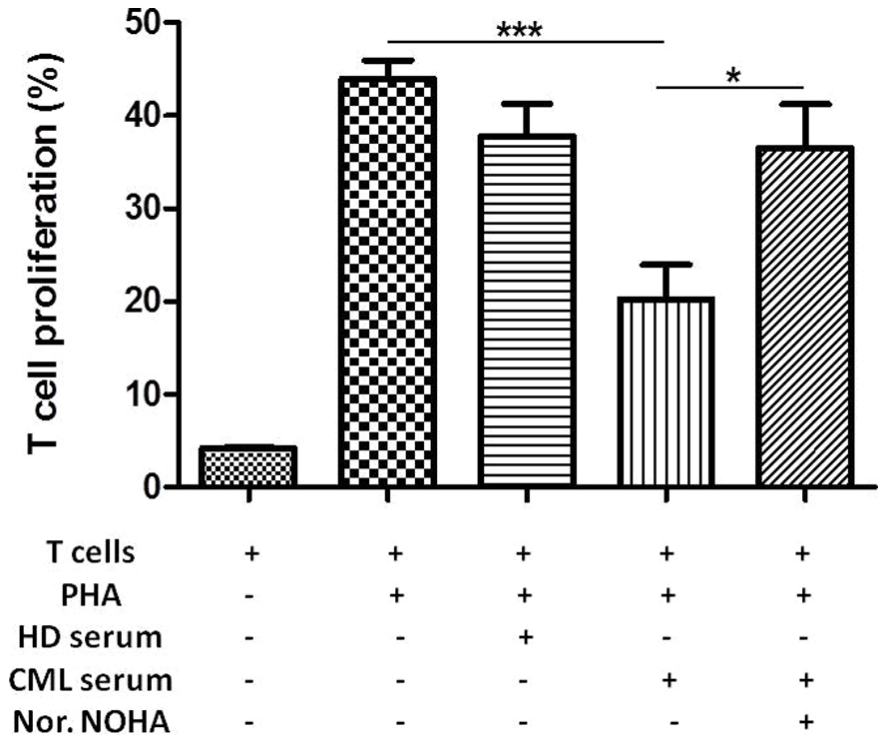
CML serum has immunosuppressive activity linked to Arg1. 8 CML sera were analyzed on 4 different HD T cells with a reduction of T cell proliferation (p<0.05).The effect was lost after addition of Nor.NOHA. Mean frequency of CD3+ CFSE ^dim^ cells ± SEM is shown.

Since CML patients at diagnosis have high levels of Arg1 in serum, subsequent experiments were designed to further understand whether or not circulating protein exerts immunosuppressive activity with CML MDSCs and PMNs. First we evaluated the effect of CML serum at different concentration on HD T cell proliferation. Since the major inhibitory effect was obtained with 20% serum (data not shown), only this concentration is reported in fig 5. After 72 h from mitogen stimulation and incubation with HD or CML serum, the percentage of proliferation of CFSE-labeled T cells was inhibited of 23,6±7,4% with CML serum compared to positive control (HD T cells plus PHA) (p<0.05). Most importantly, this inhibitory effect was lost by the addition of Nor.NOHA (an Arg1 inhibitor) (p<0.05).

## Discussion

In CML, like in other malignancies, the immune surveillance against cancer is impaired, resulting in immune escape of the malignant cells. Immunoinhibitory mechanisms such as Treg has been reported [31] and tumor cells display ligands for the PD-1inhibitory receptor [32]. MDSCs have been shown to play a central role in anti-tumor immune response in a variety of cancers including hematological malignances [33]. There are very limited data regarding MDSCs in CML. Christiansson et al. showed an increase of the Gr-MDSCs subset in CML patients that was limited to Sokal high risk patients. Since in our center most Sokal high risk patients are treated with second generation TKIs, they were not included in this study. Therefore our series comprises only 2 patients belonging to this high risk group and we cannot verify if there is a correlation between the amount of Gr-MDSC and Sokal risk. In addition, differently from our study, in Christiansson's paper cryopreserved samples were analyzed and functional experiments were not provided [32].

In the present study, evaluating the percentage of MDSCs cells in CML patients defined as CD11b+CD33+CD14-HLADR- (Gr-MDSCs) and CD14+HLADR- (Mo-MDSCs), we found that both were significantly higher at diagnosis compared to HD and decreased to normal levels after IM therapy. Since MDSCs drive the development of T-reg [21], [22], [34], in some patients we also investigated the levels of circulating T-reg cells. As it has been reported [35], [36], in CML patients T-reg resulted significantly increased in respect to HD and we found that they directly correlated with Gr-MDSCs. Similar to Gr-MDSCs, the percentage of T-reg cells decreased to the HD levels after TKI therapy, confirming the correlation with the amount of granulocytic MDSCs. MDSCs expansion in CML patients may explain the already reported defects of NK from CML patients [37] and dysfunction in antigen processing and migration of CML dendritic cells [38] since the cross-talk between MDSCs and these cells impairs their function [22].

MDSCs are characterized as immature myeloid cells (IMCs) and since CML cells mostly consist of IMCs, we hypothesized that the tumor cells themselves might be MDSCs. Analyzing the oncoprotein expression by real time PCR, both Gr-MDSCs and Mo-MDSCs expressed BCR/ABL (data not shown). Therefore, some tumor cells may be accounted for as MDSCs.

Our data also show that CML cells are characterized by high expression of Arg1 and CML Gr-MDSCs expressed higher levels of Arg1 mRNA in respect to HD. In addition, analyzing Arg1 in sera of CML patients, the levels of circulating protein resulted significantly increased at diagnosis in respect with HD and decreased during IM therapy.

Since Arg1 appears to be the major mediator of T cell suppression by both Gr-MDSCs and PMNs in cancer patients [29], [30], [39], [40], we analyzed Arg1 expression and activity in these two myeloid subsets comparing CML Gr-MDSCs to autologous PMNs. Both Arg1 protein and enzyme activity was higher in CML PMNs than Gr-MDSCs, demonstrating, for the first time, a critical role of CML PMNs on the tumor microenvironment with a potential immunosuppressive activity.

To demonstrate this suppressive capacity of CML PMNs and verify whether the population of cells with the phenotype of Gr-MDSCs that accumulates in PB of CML patients could be functionally defined as MDSCs, we determined their ability to inhibit T cell response. Both CML Gr-MDSCs and CML PMNs mediated a significantly suppression of T cell proliferation; on the contrary, no inhibitory effect was observed by Gr-MDSCs and PMNs from HD.

Since CML patients showed high levels of circulating Arg1, we also investigated whether the serum from patients at diagnosis has immunoinhibitory effects. We found a reduction of HD T cell proliferation after exposure to CML serum, but not to HD one. Inhibition of Arg1 by Nor.NOHA reversed this suppressive effect.

This current work also shows for the first time a significant overlap between Gr-MDSCs and PMNs in CML patients. In fact, even if Gr-MDSCs and PMNs are considered phenotypically and functionally different myeloid subsets, they share many common features that may lead to a critical evaluation of their relationship. In first, Gr-MDSCs show similar functions to PMNs promoting immunosuppression, angiogenesis [41], [42], invasion and metastasis [43], [44]. As PMNs [27], MDSCs acquire strong immunosuppressive activity after activation [45]. In addition, in contrast to murine Gr-MDSCs, the human Gr-MDSCs are identified by a set of antigens (CD11b, CD14, CD15, CD33, CD66b and HLADR) which are well established markers for PMNs [46], [47]. Therefore, the significant overlap between the two myeloid cell populations concerns both their function and their immunophenotype. In contrast to conventional PMNs, Gr-MDSCs are purified from the mononuclear cell fraction in density gradient of peripheral blood [48]. Despite the low percentage of CD11b+CD33+ CD14- HLADR- CD34+ cells (immature myeloid cells, IMCs), CML Gr-MDSCs are more immature compared to autologous PMNs and showed lower levels of expression of CD11b, CD15 and CD16 and lower Arg1 expression and activity (data not shown). A large number of published works are consistent with the hypothesis that tumor associated PMNs and Gr-MDSCs represent functional states of cells originating from the same cell type [48].

Nearly half of CML patients treated with imatinib who have reached durable complete molecular response are able to stop the treatment without relapse although they have a minimal amount of residual leukemia cells left [49], [50]. This implies that the immune system is able to restrain the tumor cell expansion. However, there is a lack of specific prognostic factors which could determine the restarting of the leukemic growth. There is increasing evidence suggesting that NK-cells are important in controlling the leukemic cells: increased NK-cell counts seem to correlate with the successful imatinib discontinuation [51]. It could be of interest the monitoring of MDSCs in patients who have discontinued TKI treatment in order to see if their increase could correlate with the restarting of the leukemic growth.

In conclusion our results suggest a strong potential immune escape mechanism in CML patients created by MDSCs and PMNs which control the anti-tumor reactive T cells. In addition, MDSCs should be monitored in imatinib discontinuation trials to understand their importance in relapsing patients.
